# Expression of Long Non-coding RNA in Oral Squamous Cell Carcinoma and Its Implication in Response to Radiation Therapy

**DOI:** 10.7759/cureus.84839

**Published:** 2025-05-26

**Authors:** Gurumoorthy AN, Ramesh M, Ramajayam Govindan, Akshar Aravind, Inpharasun SA, Rahul Pavshere, MaheshKumar Poomarimuthu, Essakiraja K

**Affiliations:** 1 Department of Surgical Oncology, Government Rajaji Hospital, Madurai, IND; 2 Department of Surgical Oncology, Madurai Medical College, Madurai, IND; 3 Multidisciplinary Research Unit, Madurai Medical College, Madurai, IND; 4 Multidisciplinary Research Unit, Madurai Medical College, Government Rajaji Hospital, Madurai, IND; 5 Department of Community Medicine, Government Medical College, Dindigul, Dindigul, IND

**Keywords:** lncrna, oncology, oral squamous cell carcinoma, radiotherapy, surgical oncology

## Abstract

Background

Concurrent systemic therapy with radiotherapy (RT) has been used as one of the treatment modalities in early and locally advanced tumors of the oral cavity. However, not all patients respond to radiotherapy equally because of the heterogeneity in tumor microenvironments, where long non-coding RNAs(lncRNAs) seem to have a role.

Objective

The primary objective of this study is to assess the over expression of five lncRNAs, namely, Opa interacting protein 5 (OIP5), colon cancer-associated transcript-1 (CCAT1), metastasis-associated lung adenocarcinoma transcript 1 (MALAT1), maternally expressed gene 3 (MEG3), and urothelial cancer associated 1 (UCA1) in the tissue and plasma of oral squamous cell carcinoma (OSCC) patients and correlation of this expression and response to radiation therapy. The secondary objective was to assess the feasibility of plasma lncRNA expression as a biomarker for response assessment.

Materials and methods

This is a prospective study conducted in the Department of Surgical Oncology and the Multidisciplinary Research Unit of Government Rajaji Hospital, Madurai. From 41 OSCC patients planned for radical RT, a biopsy from the primary lesion (41 test tissue samples) and a plasma sample (41 test plasma samples) were collected before RT for the detection of lncRNAs OIP5, CCAT1, MALAT1, MEG3 and UCA1, and their overexpression was compared with the control value derived from tissue samples of normal healthy oral mucosa of these OSCC patients (41 control tissue samples) and plasma samples taken from 41 healthy volunteers (41 control plasma samples), respectively. Long non-coding RNA expression was detected using the reverse transcription polymerase chain reaction (RT-PCR) technique. A fludeoxyglucose positron emission tomography/computed tomography (FDG-PET/CT) scan was performed before and three months after treatment for response assessment, and patients were classified based on response into complete responders (CR), partial responders (PR), stable disease (SD), and progressive disease (PD), as per RECIST 1.1 criteria. The differential overexpression of plasma lncRNAs was compared based on response. Statistical analysis was done using SPSS software version 21 (IBM Corp., Armonk, NY, US). The paired t-test and chi-square test were used for statistical significance.

Results

Among progressive disease (PD) patients, expression of lncRNAs OIP5 (p value 0.018) and CCAT1 (p value 0.016) in the pre-treatment plasma was significantly higher compared to complete responder (CR) patients.

Conclusion

Preoperative plasma OIP5 and CCAT1 expression are associated with radiotherapy resistance. Their role as a biomarker needs further evaluation.

## Introduction

Cancer of the oral cavity is the sixteenth most common cancer worldwide, accounting for more than 300,000 cases per year [1]. Surgery and radiotherapy (RT) are the two primary treatment modalities for oral cancer. In the early stages (I-II), patients are generally managed using a single modality, whereas in locally advanced disease (III-IV), combined modality treatment using surgery with adjuvant RT or concurrent systemic therapy with RT is used [2]. Patients treated with radiotherapy do not respond equally; this depends on clinical factors and biological factors [2]. The clinical factors may be tumor-related (like site of disease, stage, volume, nodal status, grade) or patient-related (like age, gender, haemoglobin, smoking habits) [2]. Biological factors that influence response to treatment in oral cancer are not as established as in breast cancer [2]. These biological factors are responsible for heterogeneity in the tumor microenvironment. Though many of these factors are being investigated, the role of non-coding RNAs has been of recent interest in many cancers [2,3]. The term non-coding RNA (ncRNAs) encompasses microRNA (miRNAs), long non-coding RNAs (lncRNAs), circular RNAs (circRNAs), and intronic RNAs [3]. Although ncRNAs do not encode proteins, they play a vital role in the regulation of gene expression through epigenetic, transcriptional, and post-transcriptional alterations [3]. Long non-coding RNAs are transcripts > 200 nucleotides in length without a protein-coding potential [3]. Among many lncRNAs that have been implicated in oral cancer [3], Opa interacting protein 5 (OIP5), colon cancer-associated transcript-1 (CCAT1), metastasis-associated lung adenocarcinoma transcript 1 (MALAT1), maternally expressed gene 3 (MEG3), and urothelial cancer-associated 1 (UCA1) were evaluated in this study. The primary objective of this study is to assess the overexpression of these five lncRNAs in the tissue and plasma of oral squamous cell carcinoma patients and the correlation of their plasma expression with response to radiation therapy. The secondary objective was to assess the feasibility of plasma lncRNA expression as a biomarker for response assessment. In this study, the lncRNAs OIP5, CCAT1, MALAT1, maternally expressed gene 3 (MEG3), and UCA1 were evaluated.

## Materials and methods

Participants

The current prospective study was conducted in the Department of Surgical Oncology and the Multidisciplinary Research Unit of Government Rajaji Hospital, Madurai, a state regional cancer center, from July 2022 to June 2023, where a total of 41 patients with squamous cell carcinoma of the oral cavity were included. All patients with advanced unresectable T4b disease, patients with T4a, T3, and T2 (AJCC 8th TNM) disease who were discussed in the multidisciplinary team to undergo concurrent systemic therapy with radiotherapy (RT) as the primary modality were included in this study. Oral SCC patients, with early-stage T1 and T2 and advanced-stage T3 and T4a, were planned for upfront surgery, and patients with non-squamous histology, such as adenocarcinomas and sarcomas, were excluded from the study. Recurrent tumors and patients with a previous history of RT were also excluded from the study.

Sample collection and preparation

In all patients, a biopsy of the primary lesion (41 test tissue samples) and a plasma sample (41 test plasma samples) were collected before treatment for the detection of lncRNA. Normal oral mucosal tissue from patients (41 control tissue samples) was used to derive tissue control, and plasma samples (41 control plasma samples) from healthy volunteers were used for plasma control. All samples were obtained with informed consent as per institutional ethical guidelines. All patients underwent definitive RT as the primary treatment. A fludeoxyglucose positron emission tomography/computed tomography (FDG-PET/CT) scan was performed before RT and repeated after three months of completion of RT for objective response evaluation. The expression in tissue and plasma samples was expressed as a fold change compared to controls (a value of 6 in the test tissue sample indicates the lncRNA level was elevated six times more than that of the tissue control).

LncRNA levels estimation by qPCR

Total RNA from plasma and tissue was extracted by using a TRIzol-based isolation protocol (Invitrogen Life Technologies, Carlsbad, California, USA, Catalog number 15596026). The quantity and quality of RNA were measured using the NanoDrop LITE instrument (Thermo Fisher, Carlsbad, California, USA). After that, the RNA was reverse transcribed using the high-capacity cDNA Reverse Transcription kit (Applied Biosystems, Waltham, Massachusetts, USA, Catalog number 4368814) according to the manufacturer’s protocol. The expression of candidate lncRNAs (OIP5-AS1, CCAT1, MALAT1, MEG3, UCA1) was detected by Step One RT- PCR system (Applied Biosystems) using SSO advanced universal SYBR Green supermix (Bio-Rad, Hercules, California, USA, catalog number 1725271) according to the manufacturer’s instructions. Data were normalized using internal controls (β-actin and GAPDH) expression by the comparative threshold cycle method. The relative expression levels and fold change of candidate lncRNAs were calculated using the 2 −ΔΔCt method.

Statistical analysis

All data were collected and analyses were made using SPSS software version 21 (IBM Corp., Armonk, NY, US). The paired t-test and chi-square test were used for statistical significance.

## Results

A total of 41 patients were included in this study. Except for one patient who had a T2 disease, the rest of the 40 patients (5 patients had T4b, 25 patients had T4a, and 10 patients had T3) had locally advanced disease at presentation. The most common subsites were the tongue and buccal mucosa, with 16 patients each, followed by the alveolus in 5 patients, the hard palate in 3 patients, and the floor of the mouth in 1 patient. Among the sample population, nine were female patients, and 32 were male patients. The median age of the sample was 59 (IQR 19).

Pre-treatment lncRNA evaluation

Before the initiation of treatment, lncRNA levels in the tumor tissue and plasma of patients were estimated. Table 1 shows the mean lncRNA expression in the tissue and plasma. The pretreatment tumor tissue lncRNAs OIP5, CCAT1, UCA1, and MALAT1 were overexpressed (value more than 1) in 69.29%, 70.73%, 31.70%, and 36.58% samples, respectively, as compared to the control value (value =1) derived from normal mucosal samples of patients. The pretreatment plasma lncRNAs OIP5, CCAT1, UCA1, and MALAT1 were overexpressed (value more than 1) in 51.21%, 56.1%, 14.63%, and 68.29% samples, respectively, compared to the control value (value =1) derived from plasma samples of healthy volunteers. Pretreatment MEG3 values were under-expressed (a value less than 1) in 46.34% of tissue and 29.26% of plasma samples.

**Table 1:** Pre-treatment plasma and pre-treatment tissue lncRNA levels - mean and standard deviation Opa interacting protein 5 - OIP5; colon cancer-associated transcript-1 - CCAT1; metastasis-associated lung adenocarcinoma transcript 1 - MALAT1; maternally expressed gene 3 - MEG3; urothelial cancer-associated 1 - UCA1

LncRNA	Pretreatment plasma		Pretreatment tissue	
	Mean	Standard deviation	Mean	Standard Deviation
OIP5	6.00 fold change (2^-ΔΔCT^)	15.39 fold change (2^-ΔΔCT^)	66.97 fold change (2^-ΔΔCT^)	165.19 fold change (2^-ΔΔCT^)
CCAT1	2.22 fold change (2^-ΔΔCT^)	2.76 fold change (2^-ΔΔCT^)	250.15 fold change (2^-ΔΔCT^)	717.10 fold change (2^-ΔΔCT^)
UCA1	0.81 fold change (2^-ΔΔCT^)	1.76 fold change (2^-ΔΔCT^)	38.83 fold change (2^-ΔΔCT^)	168.29 fold change (2^-ΔΔCT^)
MEG3	23.43 fold change (2^-ΔΔCT^)	69.97 fold change (2^-ΔΔCT^)	4.49 fold change (2^-ΔΔCT^)	7.65 fold change (2^-ΔΔCT^)
MALAT1	30.98 fold change (2^-ΔΔCT^)	62.35 fold change (2^-ΔΔCT^)	1.06 fold change (2^-ΔΔCT^)	1.18 fold change (2^-ΔΔCT^)

Post-treatment response evaluation

Based on the post treatment FDG-PET/CT scan taken three months after treatment, the patients were categorized into complete responders (CR), partial responders (PR), stable disease (SD) and progressive disease (PD), as per RECIST 1.1 criteria (Table 2), 10 patients had complete response and are on follow up, 16 patients had a partial response, of which 9 underwent salvage surgery, 5 were subjected to further chemotherapy, and 2 patients defaulted further treatment. Six patients fell in the stable disease category, five underwent salvage surgery, and one patient received palliative chemotherapy. The disease was progressive in nine patients, eight of whom received further cycles of chemotherapy, and one patient underwent salvage surgery.

**Table 2:** Distribution of patients based on response assessment and outcomes

Post Rt Response	Response	Salvage Surgery	Chemo Therapy	Defaulted
Complete responders	10			
Partial responders	16	9	5	2
Stable disease	6	5	1	
Progressive disease	9	1	8	

Differential expression of lncRNA based on response

The pre-treatment plasma lncRNA levels were analysed based on response to treatment. Overexpression of lncRNA varied widely within each category of response. A comparison was made between the pre-treatment lncRNA levels in the plasma of CR and PD patients. Table 3 shows the average plasma lncRNA expression based on their response. There was significant differential overexpression (higher fold change value in PD compared to CR) of OIP5 (p value 0.018, with a chi-square value of 5.625 and degree of freedom 1), CCAT1 (p value 0.016, chi-square value of 5.760, and degree of freedom 1 ), UCA1 (p value 0.047, with a chi-square value of 3.938 and degree of freedom 1) levels in the pre-treatment plasma of PD patients compared to CR patients.

**Table 3:** Pretreatment plasma lncRNA levels based on response assessment – mean and standard deviation Opa interacting protein 5 - OIP5; colon cancer-associated transcript-1 - CCAT1; metastasis-associated lung adenocarcinoma transcript 1 - MALAT1; maternally expressed gene 3 - MEG3; urothelial cancer-associated 1 - UCA1

Pre-treatment plasma	Complete Responder		Partial Responder		Stable Disease		Progressive Disease	
	Mean	Standard Deviation	Mean	Standard Deviation	Mean	Standard Deviation	Mean	Standard Deviation
OIP5	2.11 fold change (2^-ΔΔCT^)	2.12 fold change (2^-ΔΔCT^)	12.22 fold change (2^-ΔΔCT^)	23.57 fold change (2^-ΔΔCT^)	0.52 fold change (2^-ΔΔCT^)	0.56 fold change (2^-ΔΔCT^)	2.94 fold change (2^-ΔΔCT^)	3.08 fold change (2^-ΔΔCT^)
CCAT1	2.35 fold change (2^-ΔΔCT^)	2.48 fold change (2^-ΔΔCT^)	2.31 fold change (2^-ΔΔCT^)	3.22 fold change (2^-ΔΔCT^)	0.55 fold change (2^-ΔΔCT^)	0.79 fold change (2^-ΔΔCT^)	3.05 fold change (2^-ΔΔCT^)	2.94 fold change (2^-ΔΔCT^)
UCA1	0.58 fold change (2^-ΔΔCT^)	1.26 fold change (2^-ΔΔCT^)	0.92 fold change (2^-ΔΔCT^)	2.15 fold change (2^-ΔΔCT^)	0.18 fold change (2^-ΔΔCT^)	0.22 fold change (2^-ΔΔCT^)	1.29 fold change (2^-ΔΔCT^)	2.09 fold change (2^-ΔΔCT^)
MEG3	7.87 fold change (2^-ΔΔCT^)	8.09 fold change (2^-ΔΔCT^)	15.65 fold change (2^-ΔΔCT^)	32.80 fold change (2^-ΔΔCT^)	95.49 fold change (2^-ΔΔCT^)	168.35 fold change (2^-ΔΔCT^)	6.51 fold change (2^-ΔΔCT^)	6.35 fold change (2^-ΔΔCT^)
MALAT1	18.12 fold change (2^-ΔΔCT^)	32.74 fold change (2^-ΔΔCT^)	21.65 fold change (2^-ΔΔCT^)	46.01 fold change (2^-ΔΔCT^)	42.43 fold change (2^-ΔΔCT^)	58.92 fold change (2^-ΔΔCT^)	54.21 fold change (2^-ΔΔCT^)	104.91 fold change (2^-ΔΔCT^)

## Discussion

lncRNAs belong to a class of RNAs that have little or no coding potential, and by definition, they must be longer than 200 nucleotides [2,3]. They function as tumor suppressor or oncogenes in the pathogenesis and progression of various cancers. The role of lncRNA in the progression of disease and response to radiotherapy has been a recent topic of interest. Many studies have demonstrated tissue lncRNA expression in OSCC, but studies on plasma lncRNA expression are limited.

The mechanisms by which lncRNAs contribute to the pathogenesis and progression of cancer include dysregulated DNA repair, apoptosis, and immune escape [3]. They also play a role in the metastases of tumors by promoting cancer cell stemness and epithelial-mesenchymal transition [3]. Among numerous lncRNAs, OIP5-AS1, MALAT1, UCA1, CCAT1, and MEG3 have been implicated in oral squamous cell carcinoma, and these lncRNAs were included in this study.

MALAT1, which was used as a prognostic biomarker in non-small cell lung cancer [4], has also been found to be overexpressed in OSCC tissues by Liang et al. [5]. MALAT1 was overexpressed in 36.58% of tissue and 68.29% of plasma samples of patients in this study. Certain studies have shown that MALAT1 expression correlates with cervical lymph node metastasis in OSCCs [6]. MALAT1 has a role in cisplatin resistance in OSCCs [7] and radioresistance in cervical and nasopharyngeal carcinomas [8,9].

Maternally expressed gene 3 (MEG3) is an lncRNA, which is maternally imprinted and serves as a tumor suppressor gene, found in chromosome 14q32.3 [10]. It has been found to inhibit the progression of OSCC by regulating the WNT/β‑catenin signalling pathway in a study by Liu et al. [10]. Chen et al. found that MEG3 is underexpressed in OSCC tissues [11]. In our study, MEG3 was underexpressed in 46.34% and 29.26% of tissue and plasma samples, respectively.

UCA1 was overexpressed in 31.70% of tissue and 14.63% of plasma samples in our study. UCA1 lncRNA was first found in bladder cancer and located at chromosome 19p13.12 [12]. Fang et al. found that it is overexpressed in OSCC tissues [13]. UCA1 expression causes cisplatin resistance of OSCC cells [12] and radioresistance in prostate and colorectal cancers [14,15].

OIP5 is found on chromosome 15 [16]. A study by Xiao et al. found its overexpression in cisplatin-resistant OSCCs in tissue samples [17]. In our study, 69.29% of tissue and 51.21% of plasma samples overexpressed OIP5. There was a differential overexpression of OIP5 in the plasma of non-responders compared to responders to radiation therapy in our study. Zou et al. found that OIP5 causes radioresistance in colorectal cancers [18].

CCAT1 has a length of 2628 nucleotides located on chromosome 8q24. In this study, 70.73% of tissue and 56.1% of plasma samples overexpressed CCAT1. In a study by Li et al., CCAT1 tissue overexpression promoted tumor cell proliferation in oral squamous cell carcinoma [19]. In our study, there was a differential overexpression of plasma CCAT1 in progressive disease patients as compared to complete responders to radiotherapy. A similar finding by Lai et al. showed that CCAT1 was up-regulated in radioresistant breast cancer tissues [20].

Based on these results, OIP5 and CCAT1 overexpression were seen in more than half of the patients in both tissue and plasma, though with wide variation in their expression within patients and within each subset of response. They also had a significant differential overexpression in the plasma of progressive disease patients compared to complete responders to radiotherapy. UCA1 overexpression in plasma and tissue were seen in few patients in this study limiting its role as a biomarker.

There were several limitations to the study. The sample size was small, so the role of plasma lncRNA levels as a biomarker could not be established. Another limitation was the use of normal oral mucosa of patients to derive tissue control, whereas the plasma of healthy volunteers was used for plasma control, because of which the sensitivity and specificity of plasma lncRNA expression in detecting tissue overexpression could not be made out.

## Conclusions

Pretreatment tissue and plasma overexpression of OIP5 and CCAT1 are seen in oral squamous cell carcinoma patients. A higher expression of OIP5 and CCAT1 in plasma is associated with radiotherapy resistance. Their role as a biomarker in predicting response to radiotherapy needs further evaluation. Overexpression of these lncRNAs, which are associated with radiotherapy resistance in oral cancer, may guide us in deciding the definitive treatment, particularly when the patient is a surgical candidate.
